# Prediction of indirect interactions in proteins

**DOI:** 10.1186/1471-2105-7-167

**Published:** 2006-03-22

**Authors:** Peteris Prusis, Staffan Uhlén, Ramona Petrovska, Maris Lapinsh, Jarl ES Wikberg

**Affiliations:** 1 Department of Pharmaceutical Biosciences, Uppsala University, Uppsala, Sweden; 2 Linnaeus Center for Bioinformatics, Uppsala University, Uppsala, Sweden; 3 Section for Pharmacology, The University of Bergen, Bergen, Norway

## Abstract

**Background:**

Both direct and indirect interactions determine molecular recognition of ligands by proteins. Indirect interactions can be defined as effects on recognition controlled from distant sites in the proteins, e.g. by changes in protein conformation and mobility, whereas direct interactions occur in close proximity of the protein's amino acids and the ligand. Molecular recognition is traditionally studied using three-dimensional methods, but with such techniques it is difficult to predict the effects caused by mutational changes of amino acids located far away from the ligand-binding site. We recently developed an approach, proteochemometrics, to the study of molecular recognition that models the chemical effects involved in the recognition of ligands by proteins using statistical sampling and mathematical modelling.

**Results:**

A proteochemometric model was built, based on a statistically designed protein library's (melanocortin receptors') interaction with three peptides and used to predict which amino acids and sequence fragments that are involved in direct and indirect ligand interactions. The model predictions were confirmed by directed mutagenesis. The predicted presumed direct interactions were in good agreement with previous three-dimensional studies of ligand recognition. However, in addition the model could also correctly predict the location of indirect effects on ligand recognition arising from distant sites in the receptors, something that three-dimensional modelling could not afford.

**Conclusion:**

We demonstrate experimentally that proteochemometric modelling can be used with high accuracy to predict the site of origin of direct and indirect effects on ligand recognitions by proteins.

## Background

The processes of life depend on intermolecular recognition. Molecular recognition by proteins is a complex process that is determined not only by direct interactions of a protein with the interacting molecule, but also by indirect effects arising at distant sites in the proteins. Using three-dimensional (3D) structure approaches it is not straightforward to analyze long distance effects on, for example, protein conformation, mobility, and stability. Recently, a new approach using statistical analysis of protein and ligand interaction space, proteochemometrics, was developed [1,2]. It was used to model protein-peptide interactions and interactions of proteins with organic compounds [1,3-7]. Here we show its utility in predicting indirect effects in proteins.

Proteochemometrics originates from chemometrics, the mathematical methods used to analyze chemical data. Proteochemometrics aims to describe the interactions between a series of macromolecules (such as proteins) and a series of ligands. Proteochemometric models are thereby created. These models are useful for predicting the affinities of new proteins for their ligands if the new molecules fall within the description space of the protein-ligand pairs of the training data set. A proteochemometric experiment is typically described by three descriptor blocks; the ligand descriptor (*D*^*L*^*)*, protein descriptor (*D*^*P*^), and ligand-protein cross-term (*D*^*LP*^) blocks. A vector of numbers, called the ligand descriptors (*D*^*L*^*)*, characterizes each ligand *L*. Similarly, each protein *P *has its protein descriptors (*D*^*P*^). If we use a linear method of regression, the negative logarithm of ligand *L's *affinity (*pK*_*LP*_) for the protein *P *is expressed by:

pKLP=pK¯+∑iNCiDiL+∑jMCjDjP+∑iN∑jMCijDijLP     Eq. 1
 MathType@MTEF@5@5@+=feaafiart1ev1aaatCvAUfKttLearuWrP9MDH5MBPbIqV92AaeXatLxBI9gBaebbnrfifHhDYfgasaacH8akY=wiFfYdH8Gipec8Eeeu0xXdbba9frFj0=OqFfea0dXdd9vqai=hGuQ8kuc9pgc9s8qqaq=dirpe0xb9q8qiLsFr0=vr0=vr0dc8meaabaqaciaacaGaaeqabaqabeGadaaakeaacqWGWbaCcqWGlbWsdaWgaaWcbaGaemitaWKaemiuaafabeaakiabg2da9maanaaabaGaemiCaaNaem4saSeaaiabgUcaRmaaqahabaGaem4qam0aaSbaaSqaaiabdMgaPbqabaaabaGaemyAaKgabaGaemOta4eaniabggHiLdGccqWGebardaqhaaWcbaGaemyAaKgabaGaemitaWeaaOGaey4kaSYaaabCaeaacqWGdbWqdaWgaaWcbaGaemOAaOgabeaaaeaacqWGQbGAaeaacqWGnbqta0GaeyyeIuoakiabdseaenaaDaaaleaacqWGQbGAaeaacqWGqbauaaGccqGHRaWkdaaeWbqaamaaqahabaGaem4qam0aaSbaaSqaaiabdMgaPjabdQgaQbqabaaabaGaemOAaOgabaGaemyta0eaniabggHiLdaaleaacqWGPbqAaeaacqWGobGta0GaeyyeIuoakiabdseaenaaDaaaleaacqWGPbqAcqWGQbGAaeaacqWGmbatcqWGqbauaaGccaWLjaGaaCzcaiabdweafjabdghaXjabc6caUiabbccaGiabigdaXaaa@6858@

where pK¯
 MathType@MTEF@5@5@+=feaafiart1ev1aaatCvAUfKttLearuWrP9MDH5MBPbIqV92AaeXatLxBI9gBaebbnrfifHhDYfgasaacH8akY=wiFfYdH8Gipec8Eeeu0xXdbba9frFj0=OqFfea0dXdd9vqai=hGuQ8kuc9pgc9s8qqaq=dirpe0xb9q8qiLsFr0=vr0=vr0dc8meaabaqaciaacaGaaeqabaqabeGadaaakeaadaqdaaqaaiabdchaWjabdUealbaaaaa@2F45@ is the average affinity, *C*_*i*_, *C*_*j*_, and *C*_*ij *_are the regression coefficients for ligand descriptors, protein descriptors, and ligand-protein cross-terms, respectively, and *N *and *M *are the number of descriptors for ligands and proteins, respectively.

Ligand-protein cross-terms are usually defined by a new vector that is formed by multiplying each ligand descriptor with each receptor descriptor of particular ligand-receptor pairs. Hence,

DijLP=DiLDjP     Eq. 2
 MathType@MTEF@5@5@+=feaafiart1ev1aaatCvAUfKttLearuWrP9MDH5MBPbIqV92AaeXatLxBI9gBaebbnrfifHhDYfgasaacH8akY=wiFfYdH8Gipec8Eeeu0xXdbba9frFj0=OqFfea0dXdd9vqai=hGuQ8kuc9pgc9s8qqaq=dirpe0xb9q8qiLsFr0=vr0=vr0dc8meaabaqaciaacaGaaeqabaqabeGadaaakeaacqWGebardaqhaaWcbaGaemyAaKMaemOAaOgabaGaemitaWKaemiuaafaaOGaeyypa0Jaemiraq0aa0baaSqaaiabdMgaPbqaaiabdYeambaakiabdseaenaaDaaaleaacqWGQbGAaeaacqWGqbauaaGccaWLjaGaaCzcaiabdweafjabdghaXjabc6caUiabbccaGiabikdaYaaa@41ED@

and then

pKLP=pK¯+∑iNCiDiL+∑jMCjDjP+∑iNDiL[∑jMCijDjP]     Eq. 3
 MathType@MTEF@5@5@+=feaafiart1ev1aaatCvAUfKttLearuWrP9MDH5MBPbIqV92AaeXatLxBI9gBaebbnrfifHhDYfgasaacH8akY=wiFfYdH8Gipec8Eeeu0xXdbba9frFj0=OqFfea0dXdd9vqai=hGuQ8kuc9pgc9s8qqaq=dirpe0xb9q8qiLsFr0=vr0=vr0dc8meaabaqaciaacaGaaeqabaqabeGadaaakeaacqWGWbaCcqWGlbWsdaWgaaWcbaGaemitaWKaemiuaafabeaakiabg2da9maanaaabaGaemiCaaNaem4saSeaaiabgUcaRmaaqahabaGaem4qam0aaSbaaSqaaiabdMgaPbqabaaabaGaemyAaKgabaGaemOta4eaniabggHiLdGccqWGebardaqhaaWcbaGaemyAaKgabaGaemitaWeaaOGaey4kaSYaaabCaeaacqWGdbWqdaWgaaWcbaGaemOAaOgabeaaaeaacqWGQbGAaeaacqWGnbqta0GaeyyeIuoakiabdseaenaaDaaaleaacqWGQbGAaeaacqWGqbauaaGccqGHRaWkdaaeWbqaaiabdseaenaaDaaaleaacqWGPbqAaeaacqWGmbataaaabaGaemyAaKgabaGaemOta4eaniabggHiLdGcdaWadaqaamaaqahabaGaem4qam0aaSbaaSqaaiabdMgaPjabdQgaQbqabaGccqWGebardaqhaaWcbaGaemOAaOgabaGaemiuaafaaaqaaiabdQgaQbqaaiabd2eanbqdcqGHris5aaGccaGLBbGaayzxaaGaaCzcaiaaxMaacqWGfbqrcqWGXbqCcqGGUaGlcqqGGaaicqqGZaWmaaa@6B84@

By using *Eq. 3*, the selectivity, *S_LAB_*, between protein *A *and protein *B *for some particular ligand *L *can be expressed as:

SLAB=pKLA−pKLB=∑jMCj(DjA−DjB)+∑iNDiL[∑jMCij(DjA−DjB)]     Eq. 4
 MathType@MTEF@5@5@+=feaafiart1ev1aaatCvAUfKttLearuWrP9MDH5MBPbIqV92AaeXatLxBI9gBaebbnrfifHhDYfgasaacH8akY=wiFfYdH8Gipec8Eeeu0xXdbba9frFj0=OqFfea0dXdd9vqai=hGuQ8kuc9pgc9s8qqaq=dirpe0xb9q8qiLsFr0=vr0=vr0dc8meaabaqaciaacaGaaeqabaqabeGadaaakeaacqWGtbWudaWgaaWcbaGaemitaWKaemyqaeKaemOqaieabeaakiabg2da9iabdchaWjabdUealnaaBaaaleaacqWGmbatcqWGbbqqaeqaaOGaeyOeI0IaemiCaaNaem4saS0aaSbaaSqaaiabdYeamjabdkeacbqabaGccqGH9aqpdaaeWbqaaiabdoeadnaaBaaaleaacqWGQbGAaeqaaaqaaiabdQgaQbqaaiabd2eanbqdcqGHris5aOWaaeWaaeaacqWGebardaqhaaWcbaGaemOAaOgabaGaemyqaeeaaOGaeyOeI0Iaemiraq0aa0baaSqaaiabdQgaQbqaaiabdkeacbaaaOGaayjkaiaawMcaaiabgUcaRmaaqahabaGaemiraq0aa0baaSqaaiabdMgaPbqaaiabdYeambaaaeaacqWGPbqAaeaacqWGobGta0GaeyyeIuoakmaadmaabaWaaabCaeaacqWGdbWqdaWgaaWcbaGaemyAaKMaemOAaOgabeaaaeaacqWGQbGAaeaacqWGnbqta0GaeyyeIuoakmaabmaabaGaemiraq0aa0baaSqaaiabdQgaQbqaaiabdgeabbaakiabgkHiTiabdseaenaaDaaaleaacqWGQbGAaeaacqWGcbGqaaaakiaawIcacaGLPaaaaiaawUfacaGLDbaacaWLjaGaaCzcaiabdweafjabdghaXjabc6caUiabbccaGiabisda0aaa@7388@

If a region *U *of a protein is described by the set of descriptors, *V*, then the contribution to the selectivity, SLABU
 MathType@MTEF@5@5@+=feaafiart1ev1aaatCvAUfKttLearuWrP9MDH5MBPbIqV92AaeXatLxBI9gBaebbnrfifHhDYfgasaacH8akY=wiFfYdH8Gipec8Eeeu0xXdbba9frFj0=OqFfea0dXdd9vqai=hGuQ8kuc9pgc9s8qqaq=dirpe0xb9q8qiLsFr0=vr0=vr0dc8meaabaqaciaacaGaaeqabaqabeGadaaakeaacqWGtbWudaqhaaWcbaGaemitaWKaemyqaeKaemOqaieabaGaemyvaufaaaaa@3274@, by *U *between proteins *A *and *B *for ligand *L *is obtained by:

SLABU=∑j∈VKCj(DjA−DjB)+∑iNDiL[∑j∈VKCij(DjA−DjB)]     Eq. 5
 MathType@MTEF@5@5@+=feaafiart1ev1aaatCvAUfKttLearuWrP9MDH5MBPbIqV92AaeXatLxBI9gBaebbnrfifHhDYfgasaacH8akY=wiFfYdH8Gipec8Eeeu0xXdbba9frFj0=OqFfea0dXdd9vqai=hGuQ8kuc9pgc9s8qqaq=dirpe0xb9q8qiLsFr0=vr0=vr0dc8meaabaqaciaacaGaaeqabaqabeGadaaakeaacqWGtbWudaqhaaWcbaGaemitaWKaemyqaeKaemOqaieabaGaemyvaufaaOGaeyypa0ZaaabCaeaacqWGdbWqdaWgaaWcbaGaemOAaOgabeaaaeaacqWGQbGAcqGHiiIZcqWGwbGvaeaacqWGlbWsa0GaeyyeIuoakmaabmaabaGaemiraq0aa0baaSqaaiabdQgaQbqaaiabdgeabbaakiabgkHiTiabdseaenaaDaaaleaacqWGQbGAaeaacqWGcbGqaaaakiaawIcacaGLPaaacqGHRaWkdaaeWbqaaiabdseaenaaDaaaleaacqWGPbqAaeaacqWGmbataaaabaGaemyAaKgabaGaemOta4eaniabggHiLdGcdaWadaqaamaaqahabaGaem4qam0aaSbaaSqaaiabdMgaPjabdQgaQbqabaaabaGaemOAaOMaeyicI4SaemOvayfabaGaem4saSeaniabggHiLdGcdaqadaqaaiabdseaenaaDaaaleaacqWGQbGAaeaacqWGbbqqaaGccqGHsislcqWGebardaqhaaWcbaGaemOAaOgabaGaemOqaieaaaGccaGLOaGaayzkaaaacaGLBbGaayzxaaGaaCzcaiaaxMaacqWGfbqrcqWGXbqCcqGGUaGlcqqGGaaicqqG1aqnaaa@6E58@

Accordingly, we can localize regions in a protein that afford selectivity (i.e., functionality difference between protein pairs) for a particular ligand by applying *Eq. 5*. A region can be a subsequence, a 3D molecular interaction field, a single amino acid, or even a physicochemical property of an individual amino acid, and is only restricted by the way the descriptors are assigned to the proteins [1-7]. Since *Eq. 5 *places no restriction how far in space a ligand is located from a region in a protein, proteochemometrics is useful to predict indirect interactions in proteins.

## Results

### Design and testing of multiple chimeric melanocortin receptor library

It is necessary to have modifications of both the proteins and ligands, preferably both in the form of a series (i.e., "libraries"), in order to use *Eq. 5*. Here we used a library of multiple chimeric melanocortin receptors [7]. Briefly, the library was created from four natural melanocortin receptors (MC_1_, MC_3–5_). Each receptor was divided into five sequence fragments (S1–S5) and multiple chimeric receptors were then obtained by systematically shuffling the fragments. In order to maximize the chemical space information of the receptors, while keeping the number of experiments as low as possible, statistical molecular design was applied to properly select the sequence fragments [7-10]. The entire receptor library comprised 18 receptors and was tested for its interaction with the native melanocortin ligand, α-MSH and the synthetic MSH analogues NDP-MSH and [^125^I]-NDP-MSH (see Table 1).

### Proteochemometrics modelling

Interpretation of *Eq.5 *is dependent on our description of the proteins. Here two proteochemometrics models were created. One was based on a binary description of the proteins. The other used physicochemical descriptions of amino acids located inside transmembrane regions with presumed proximity to possible ligand binding cleft(s) according to the x-ray structure of bovine rhodopsin [7,11,12]. The binary model comprised information on the extent to which segments S1–S5 are involved in the selective recognition of ligands by the receptors, while the model based on physicochemical descriptions of amino acids comprised information on the contributions of single amino acids. Both models performed excellently in state-of-the-art model validations (see Table 2).

### Prediction of ligand recognition

**Figure 1:**
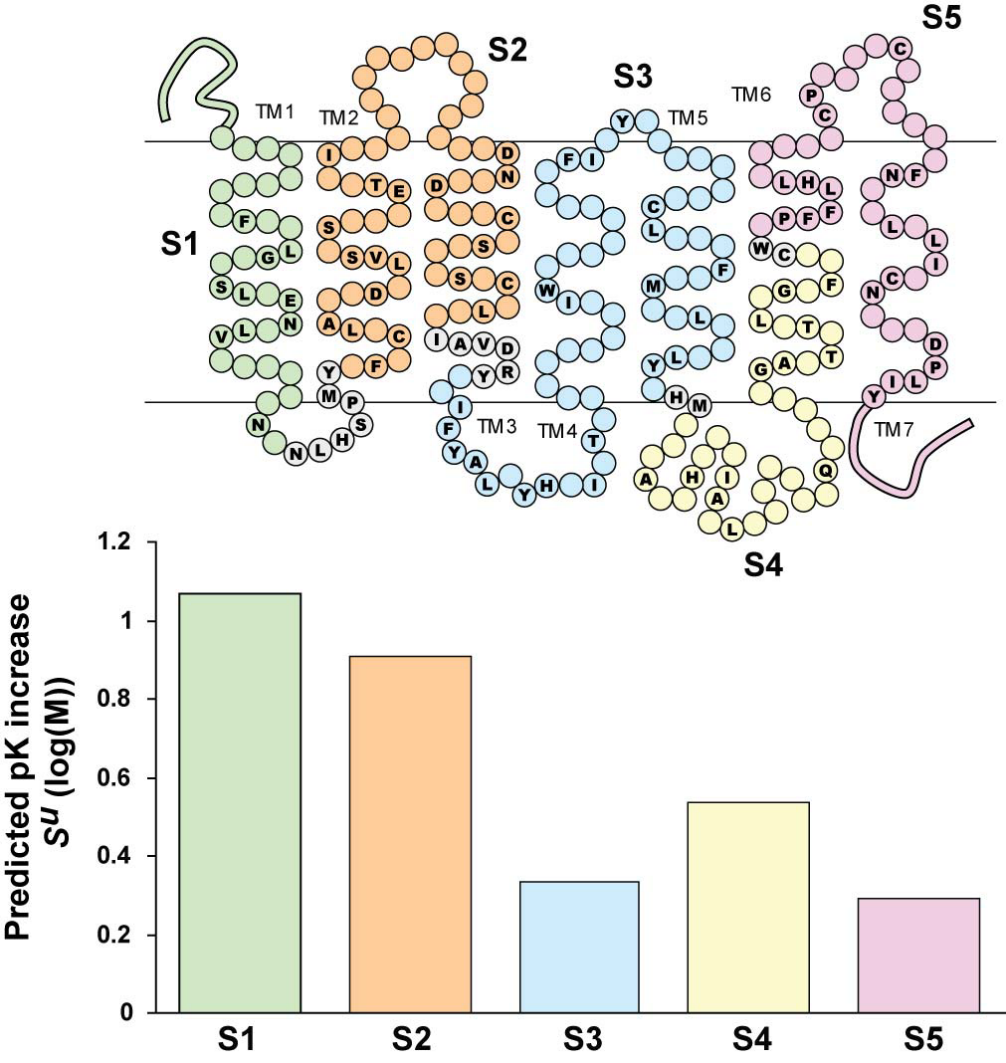
**Increase in affinity of α-MSH predicted from the binary proteochemometric model**. Shown is the increase in affinity *S*^*U *^(log(M)) for α-MSH afforded by swapping each of segments S1 – S5 in the MC_4 _receptor with the corresponding segment in the MC_1 _receptor as predicted from the binary proteochemometrics model.

The models were used to compute selectivity recognition maps for all melanocortin receptor pairs for the α-MSH hormone (see Tables 3 and 4). α-MSH is recognized by MC receptors, albeit it binds with more than 1000 times higher affinity to the MC_1 _receptor than to the MC_4 _receptor. The recognition map derived from the binary model predicted that segments S1, S2, and S4 play major roles for the α-MSH MC_1_/MC_4 _selectivity, while S3 and S5 contribute only a little (Fig. 1). The involvement of S1 and S2 was expected, as these regions possess amino acids close to the ligand recognition site according to 3D modelling [11]. However, the whole S4 region was located farther away, and its involvement was therefore more surprising. The model based on amino-acid physicochemical properties in the transmembrane regions indicated that three amino acids in S1, five in S2, one in S4, and one in S5 caused the MC_1_/MC_4 _receptor selectivity (see Table 4).

### Verification of predictions using mutagenesis

**Figure 2:**
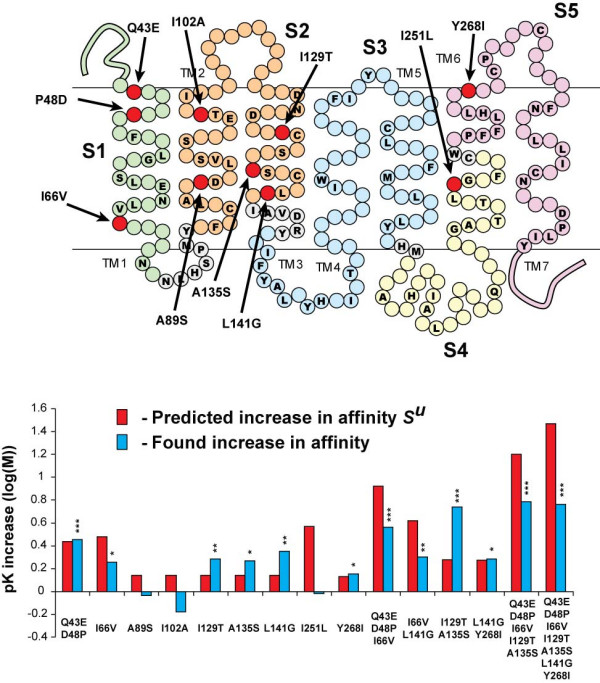
**Predicted and experimentally determined increase in α-MSH affinity for site-directed mutants**. Predicted and experimentally determined increase in affinity (i.e., computed SU and measured pK increase, log(M), respectively) for α-MSH afforded by point mutations in the MC_4 _receptor. Shown by red bars is the change in affinity predicted by the model utilizing physicochemical descriptions of amino acids of the receptors' transmembrane regions and facing potential ligand binding clefts to be afforded by the indicated point mutations (i.e., the predicted increase in affinity that should be gained by exchanging the indicated amino acids in the MC_4 _receptor with the corresponding ones in the MC_1 _receptor). Shown in blue bars is the experimentally determined change in affinity for these mutations vs. the wild-type MC_4 _receptor. Significance (nonparametric Wilcoxon Rank Sum statistical test [29]) denoted as follows: * p <0.05; ** p < 0.005; *** p < 0.0005.

In order to assess the predictions for the ten amino acid positions predicted with by the model based on physicochemical properties of amino-acids located inside the receptors transmembrane regions, they were subjected to single, double, triple, pentuple, and heptuple mutations in the MC_4 _receptor, replacing them with the corresponding amino acids in the MC_1 _receptor. Measurements taken from the mutants showed that seven positions gave the expected increase in affinity for α-MSH (see Fig. 2, Table 5). Combining amino acid substitutions also gave higher increases in affinity than did the single point mutations (see Fig. 2, Table 5).

However, three of the ten positions (A89S, I102A and I251L) did not show the predicted increase in affinity (see Fig. 2, Table 5). The failure of the A89S and I102A mutations to increase the affinity of the MC_4 _receptor can be explained by the presence of several amino acid positions in the protein library that show the same or similar variability (i.e., they co-vary). For example, positions A89 and A135 (numbered according to the MC_4 _receptor) both reside in the same segment and are serines in the MC_1 _receptor and alanines in the MC_3_, MC_4_, and MC_5 _receptors. Such co-varying sequence positions gain equal importance in a proteochemometric model, even when some of them are not responsible for the explained activity. In the present library, mutations A89S, I102A, I129T, A135S, and L141G represent co-varying amino acid positions. The failure of the A89S and I102A mutations to cause the predicted increase in affinity may thus be explained on the basis of co-variance, where the actual effect is caused by mutations I129T, A135S, and L141G. In fact, the sum of the experimentally determined affinity increase by mutations A89S, I102A, I129T, A135S, and L141G (pKi = 0.69) closely agrees with that predicted from the model (*S*^*U *^= 0.56).

**Figure 3:**
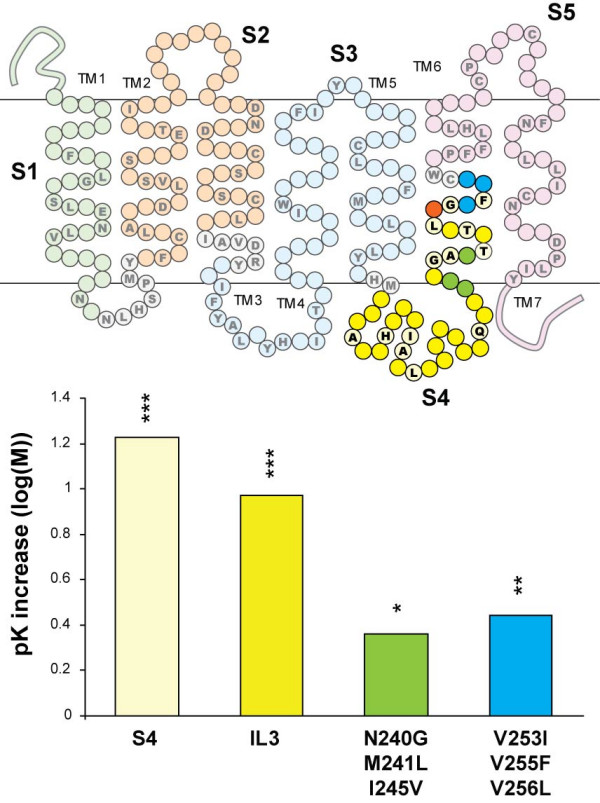
**Experimentally determined increase in α-MSH affinity by mutations in segment *S4***. Experimentally determined increase in affinity (pK) afforded by mutations N240G/M241L/I245V (indicated in green), and V253I/V255F/V256L (indicated in blue) (i.e., exchanging amino acid residues in the MC_4 _receptor with the corresponding residues in the MC_1 _receptor), and swapping intracellular loop 3 (IL3, indicated in dark yellow) in the MC_4 _receptor with the corresponding in the MC_1 _receptor. Also shown is the increase in affinity for the whole S4 segment (S4, indicated in pale yellow). Significance (nonparametric Wilcoxon Rank Sum statistical test [29]) denoted as indicated in the legend to Fig. 2.

However, the failure of mutation I251L to increase affinity could not be explained by co-variances of amino acids within the model. Accordingly the predicted effect must originate from amino acids actually co-varying in the library but excluded from the modelling in the selections of amino acids based on 3D structure. Three such possible excluded positions partially co-varying with I251 are found in the midst of TM6, three are found towards the intracellular face of the lipid bi-layer in TM6, and several are present in the third intracellular loop. In the 3D structure, these amino acids are obviously located very far from any eventual binding pocket for α-MSH. To verify the prediction that these positions are responsible for the 'missing' affinity they were mutated separately and in combinations (see Fig. 3, Table 5). Although all mutations afforded an increase in affinity, most of the increase (~12 -fold) was afforded by swapping the entire third intracellular loop (see Fig. 3, Table 5). (Mutating all of these positions together afforded an ~20-fold increase in affinity).

## Discussion

**Figure 4:**
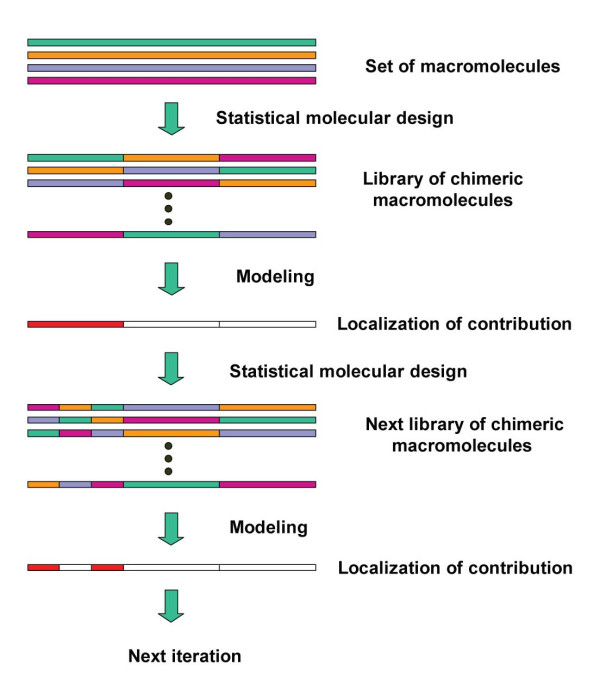
**Outline of the mapping of direct and indirect interactions in molecular recognition using proteochemometrics**. Outline of a general procedure for mapping molecular recognition by biomacromolecules. Initially, sets of wild-type macromolecules are identified. Using statistical molecular design a library is then created from the wild-type macromolecules. The library can be selected from the wild-type molecules if the initial collection contains sufficient chemical variation. Chemical variation may also be introduced artificially by mutagensis. Shuffling sequence fragments can then be used to create a library, where three or more segments and three or more macromolecules are used as the starting point. After evaluating the interaction of the library with a suitable library of ligands of interest, a proteochemometric model can be created. This model may be used to localize the regions in each macromolecule that contribute to the selectivity of each particular ligand evaluated. It may happen that it is not possible to unambiguously localize individual amino acids within a particular region, due to co-varying amino acid positions in the macromolecular library. In that case, an extension of the library can be made in order to resolve the ambiguity.

The experimental data demonstrate the utility of proteochemometrics for mapping ligand recognition. Using it seven amino-acid positions and the third intracellular loop were identified as accounting for most of the difference in affinity of the MC_1 _and MC_4 _receptor for α-MSH. The ability to localize a distant effect in a protein distinguishes proteochemometrics from other approaches to mapping molecular recognition. A general scheme for mapping ligand recognition using proteochemometrics is outlined in Fig. 4. The protein library might initially be collected from wild-type proteins, or created by constructing multiple chimeric proteins, as performed in this study. Applying experimental design could, in both cases, substantially reduce the number of entities that need to be constructed and tested without detrimentally compromising the information gained [2]. The application of proteochemometric modelling to the data creates a selectivity recognition map for the proteins and ligands. Analyzing the map (*e.g.*, using Eq. 5) reveals the regions in the proteins involved in recognition of the ligands in a data-set. Regions of high interest can then be identified and analyzed further by extending the library, if necessary, in order to remove ambiguities due to co-variances (Fig. 4). In the selection of the regions for mutations the present study shows that also distant effects on protein function should be taken into account. Overall the approach would lead to a substantial gain in information at reduced experimental effort

Mapping molecular recognition with proteochemometrics obtains different information than 3D-structure-based methods. The latter generally reveal where one particular ligand touches one particular receptor in one of many bound states. Proteochemometrics, by contrast, reveals regions of biomacromolecules that influence the selectivity of their ligand binding, and it has the capacity to predict effects arising from distant sites in a protein. The information obtained is only partially overlapping and, therefore, the approaches complement each other. The information gained in proteochemometrics relates to protein function and has direct utility for changing the function of a protein in a desired direction by mutation, in *a priori *protein engineering, or by altering the structure of a chemical entity and improving its interaction with binding sites, in *a priori *drug design [2].

## Conclusion

In the present work we have presented a theoretical basis for the use of proteochemometrics to assess direct and indirect interactions in proteins, and we verified its utility to this end experimentally. We have also outlined a general strategy to afford cost effective mapping of molecular recognition using proteochemometrics and indicate important differences of proteochemometric mapping of ligand recognitions compared to traditional three-dimensional approaches. We propose that proteochemometrics can be used as a complement to classical 3D based methods to the study of ligand recognition, or even as the prime choice, in particular when three-dimensional protein structures are not available. Moreover, since proteochemometrics can be used to map both direct and indirect interactions, it may find a more general use in e.g. protein engineering and mapping protein function.

## Methods

### Receptor clones and multichimeric receptors

The coding sequences of the MC_1 _and MC_5 _receptors were cloned earlier [13,14]. The coding sequences of the MC_3 _and MC_4 _receptor were gifts of Dr. Ira Gantz [15,16]. Multichimeric MC_1,3–5 _receptors were constructed so that each receptor was divided into three regions, with each region being replaced with a corresponding region from one of the four wild-type MC_1,3–5 _receptors. Swapping all segments from the four receptors would have created a library with an extensive number of combinations, but this would have been impractical. We applied a statistical molecular design, which combines statistical approaches to maximize information gained from a minimal number of experiments. We used a D-optimal design, which could approximate all combinations of the melanocortin receptor regions making up 16 receptors, of which 4 were wild-type and 12 were multiple chimeric receptors. The multiple chimeric receptors were constructed in two sets, the F- and S-sets. The divisions were made at the end of the third and fifth transmembrane segments for the F-set (9 receptors of the 12 designed could be obtained), and at the beginning of the second and middle of the sixth transmembrane segments for the S-set (5 receptors were thus obtained, completing the library according to the design). Thus, the library came to include a total of 18 receptors: 14 that were multiple chimeric (in five segments) and four wild-type MC_1,3–5 _receptors. We have previously reported a full account of the construction of this receptor library [7]. Moreover, full accounts of the theory and applicability of statistical molecular design have also been reported previously [8-10].

### Site-directed mutagenesis

Selected individual amino acids in the MC_4 _receptor were exchanged for the corresponding amino acids of the MC_1 _receptor, using mutation-inducing primers and PCR [17]. Eight single mutants (I66V, A89S, I102A, I129T, A135S, L141G, I251L, and Y268I), a double mutant (Q43E/P48D), and two triple mutants (N240G/M241L/I245V, V253I/V255F/V256L), were created. Some of these mutants were then used as templates for constructing mutants with multiple mutations, yielding I66V/L141G, I129T/A135S, L141G/Y268I, Q43E/P48D/I66V, Q43E/P48D/I66V/I129T/A135S, and Q43E/P48D/I66V/I129T/A135S/L141G/Y268I. We also manufactured two chimeric receptors in which subsequences of the MC_4 _receptor were replaced by the corresponding subsequences of the MC_1 _receptor. First we constructed a receptor ("S4 chimeric receptor") in which the entire S4 segment in the MC4 receptor was replaced with that from the MC_1 _receptor. Another receptor ("IL3 chimeric receptor") comprised the MC_4 _receptor with intracellular loop 3 replaced with the corresponding loop of the MC_1 _receptor.

### Peptides and radioligand

Peptide ligands α-MSH (Ac-SYSMGHFRWGKPV-NH_2_) and NDP-MSH ([Tyr^2^, Nle^4^, D-Phe^7^]-α-MSH) were synthesized using standard solid phase peptide synthesis and purified by HPLC; their correct molecular weights were verified by mass spectrometry. [^125^I]-NDP-MSH ([^125^I-Tyr^2^, Nle^4^, D-Phe^7^]-α-MSH) was prepared in radiochemically pure form by custom iodination at EuroDiagnostica AB, Sweden.

### Receptor expression and radioligand binding

COS-1 cells were grown in Dulbecco's modified Eagle's medium with 10% fetal calf serum. Eighty-percent confluent cultures were transfected in 100-mm dishes with the DNA constructs (10 μg DNA per dish, mixed with liposomes, as described [18]. Twelve to sixteen hours after transfection, the serum-free medium was replaced with growth medium and the cells were cultivated for approximately 48 hours, then scraped off, centrifuged, and used for radioligand binding. Dissociation constants (K_d_) for [^125^I]-NDP-MSH were estimated for all the receptors by radioligand binding saturation as described [7,18]. Inhibition constants (K_i_) for α-MSH and NDP-MSH were then estimated in competition with [^125^I]-NDP-MSH using established procedures [7,18]. Obtained pK values (i.e., the negative logarithms of the K_i _and K_d _values) are listed in Tables 1 and 5.

### Numerical descriptions of receptors for proteochemometric modelling

Two types of descriptions were used for the receptors, namely a binary description and a description based on physicochemical descriptions of amino acids. For the binary description, four binary numbers described each region, with each number corresponding to one receptor subtype. Each of these descriptors was assigned a value of +1 if the region was taken from descriptors' corresponding receptor subtype, otherwise it was assigned the number -1 [7].

The receptor descriptions based on physicochemical descriptions of amino acids at 38 selected sequence positions was used as described [7]. These positions were selected from a three-dimensional model of the transmembrane regions of the MC_1 _receptor.

Using the crystal structure coordinates of rhodopsin as a template [12], the model was derived by replacing the side chains with the corresponding side chains of the MC_1 _receptor, using the alignments of the GPCRDB database [19] and the SCWRL program [20]. Only amino acids pointing in the direction opposite the lipid bilayer were considered. This led to a considerable reduction in the number of co-varying sequence positions considered in the proteochemometric modelling, albeit with the obvious risk of excluding important positions. The selected positions are listed in Table 4.

Each position was coded by using five principal components derived from 26 physicochemical properties of amino acids, so called z-scales [21]. These z-scales represent hydrophobicity, steric properties, polarizability (z1–z3), polarity, and electronic effects (z4, z5). The data for the 38 × 5 = 190 descriptors obtained were compressed by applying principal component analysis (PCA) [22] to each of the five segments of the receptor library, which yielded in total 15 descriptors, with three descriptors for each segment. Prior to PCA, the z-scale descriptors had been scaled to unit variance [23]. Principal component analysis was performed using the Simca-P program [23].

### Numerical description of peptides for proteochemometric modelling

The three peptide ligands used showed limited structural variation and were assigned two binary descriptors termed ***Y ***and ***F***, using the Free-Wilson approach [24]. ***Y ***was set to +1 if the Tyr^2 ^residue of the peptides was iodinated; otherwise it was set to -1. Thus, ligand descriptor ***Y ***distinguished between [^125^I]-NDP-MSH and the other two peptides. The descriptor ***F ***was set to +1 if Phe^7 ^was in the D-conformation and there was an Nle at position 4; otherwise it was set to -1. Thus, ligand descriptor F distinguished between α-MSH and the other two ligands. We also included the cross-term formed between the peptide descriptors ***Y ***and ***F ***(termed Y*F). This cross-term distinguishes NDP-MSH from α-MSH and [^125^I]-NDP-MSH. The peptide ligand description is summarized in Table 6.

### Numerical description of binding experiments and proteochemometric modelling

In each binding experiment, the receptor-peptide combination was described using the above receptor and peptide descriptors, and by computing receptor-ligand cross-terms using *Eq. 2*, each cross-term being calculated as the product of one peptide and one receptor descriptor. Prior to calculating cross-terms, all descriptors were mean-centred and scaled to unit variance [23]. In order to account for differences in the number and mutual correlation of each descriptor type, the peptide descriptors, receptor descriptors, and cross-terms, block scaling was applied. All descriptors and cross-terms were mean centred and scaled to unit variance prior to block scaling [23]. Descriptors were finally correlated to the pK values using partial least squares projection to latent structures (PLS) regression [22]. PLS modelling was done using Simca-P [23].

### Validation of modelling

The goodness-of-fit was estimated by the correlation coefficient R^2^ and root mean square error (RMSE) [23]. Models were further validated using cross-validation (CV) [25,26], validation by response permutations [27], and validation by external prediction.

In cross-validation, one divides the data into several fractions. Seven were used in this study. Each fraction is repeatedly excluded once and then predicted from the model developed on the remaining data. The goodness of prediction of the CV is assessed by the Q^2 ^measure [23].

In response permutation validation, many models are created using randomly permuted response data. Twenty permutations were used here. For each permuted model, the R^2^, Q^2^, and correlation coefficients between the original and permuted response values are estimated. The correlation coefficients are plotted against the R^2 ^and Q^2 ^values. The two corresponding linear correlation lines are estimated, one for R^2 ^and one for Q^2^, and the intercepts iR^2 ^and iQ^2 ^of the two regression lines with the zero correlation coefficient line are calculated [5,23]. These intercept values indicate the R^2 ^and Q^2 ^of random response data. For example, a negative Q^2 ^intercept shows that it is not possible to obtain predictive models with random data, and indicates that a high Q^2 ^value of the original model is not obtained by pure chance.

External prediction assesses a model's stability when a substantial fraction of the data is excluded (e.g., more than one-third). External prediction may aim to predict the properties of new entities, in other words, entities that are entirely excluded from the data set. In the present case we predicted pKs for the S-set receptors using only data for F-set receptors. The goodness of external prediction was assessed by the external Q^2 ^(eQ^2^) value [5]. Further details on these model validation approaches and how to interpret their results have been previously reported [28].

### Computation of the selectivity contribution of amino acids

The contributions of individual amino acids to the selectivity of peptide binding between pairs of receptors were computed using *Eq. 5*. In order to apply *Eq. 5*, the regression coefficients for the individual z-scales of the receptor's amino acids were computed from the corresponding PLS and PCA models. Nine amino acids (Q43, P48, I66, A89, I102, I129, L141, I251, Y268, according to the numbering in the MC_4 _receptor) contributed most to the selectivity of α-MSH (see Table 4) and were selected for site-directed mutagenesis. Moreover, upon analyzing the 3D model of the MC_1 _receptor it was deemed that S83 (corresponding to A89 in the MC_4 _receptor) had a strong H-bond interaction with S130 (A135 in the MC_4 _receptor). Since the amino acid positions A89/A135 (MC_4 _receptor numbering) showed identical co-variance in the receptor library, A135 was also selected for site-directed mutagenesis.

### Statistical tests

The distribution of measured affinity for MC_4 _receptor and mutant receptors presented here as well as its logarithm values (pK) did not correspond to normal distribution. Therefore we decided to use nonparametric Wilcoxon Rank Sum statistical test [29] to verify the hypothesis that affinity for corresponding mutant receptor differs from wild-type MC_4 _receptor. The test was performed using R program [30].

## Authors' contributions

PP did most of modeling, data analysis and interpretation and manuscript preparation work. SU was responsible for all molecular biology work. RP assisted SU as well as performed pharmacological measurements. ML gave significant intellectual contribution for modeling, data analysis and interpretation. JESW was the major initiator of the project and supervisor of it and he contributed significantly to drafting the manuscript.

## Figures and Tables

**Table 1:** Affinities of multiple chimeric melanocortin receptors. Affinities (pK ± standard deviation, SD) of multiple chimeric melanocortin receptors for α-MSH, NDP-MSH and [^125^I]-NDP-MSH determined by radioligand binding.

N^o^	Name	Set	Parts			pK		
			
			A	B	C	^125^I-NDP-MSH	NDP-MSH	α-MSH
1	MC_1_	wt.	MC_1_	MC_1_	MC_1_	9.96^a^	10.34^a^	9.68^a^
2	MC_3_	wt.	MC_3_	MC_3_	MC_3_	9.40^a^	9.31^a^	7.27^a^
3	MC_4_	wt.	MC_4_	MC_4_	MC_4_	8.64^a^	8.73^a^	5.69^a^
4	MC_5_	wt.	MC_5_	MC_5_	MC_5_	8.54^a^	8.58^a^	5.30^a^
5	F134	F	MC_1_	MC_3_	MC_4_	8.58 ± 0.17	10.37 ± 0.01	7.70 ± 0.14
6	F153	F	MC_1_	MC_5_	MC_3_	8.88 ± 0.16	10.38 ± 0.12	7.80 ± 0.56
7	F354	F	MC_3_	MC_5_	MC_4_	8.80 ± 0.16	8.66 ± 0.48	5.72 ± 0.65
8	F413	F	MC_4_	MC_1_	MC_3_	8.62 ± 0.16	8.64 ± 0.16	6.21 ± 0.42
9	F435	F	MC_4_	MC_3_	MC_5_	8.96 ± 0.08	8.81 ± 0.14	6.39 ± 0.40
10	F451	F	MC_4_	MC_5_	MC_1_	9.06 ± 0.31	8.54 ± 0.26	6.33 ± 0.43
11	F514	F	MC_5_	MC_1_	MC_4_	9.19 ± 0.21	8.45 ± 0.11	5.11 ± 0.14
12	F531	F	MC_5_	MC_3_	MC_1_	8.67 ± 0.48	10.85 ± 0.28	5.33 ± 0.08
13	F543	F	MC_5_	MC_4_	MC_3_	9.24 ± 0.25	9.51 ± 0.50	6.39 ± 0.50
14	S134	S	MC_1_	MC_3_	MC_4_	9.30 ± 0.26	9.98 ± 0.16	9.02 ± 0.22
15	S354	S	MC_3_	MC_5_	MC_4_	8.81 ± 0.18	8.62 ± 0.37	4.88 ± 0.25
16	S451	S	MC_4_	MC_5_	MC_1_	9.06 ± 0.31	9.35 ± 0.36	6.10 ± 0.16
17	S514	S	MC_5_	MC_1_	MC_4_	9.19 ± 0.21	10.04 ± 0.31	7.31 ± 0.38
18	S531	S	MC_5_	MC_3_	MC_1_	8.96 ± 0.35	9.18 ± 0.65	7.57 ± 0.19

^a^Data for MC_1_, MC_3_, MC_4_, and MC_5 _taken from [31].

**Table 2:** Performance of proteochemometric models. Performance of proteochemometric models derived using wild-type and multiple chimeric receptors interacting with melanocortins, α-MSH, NDP-MSH and [^125^]-NDP-MSH. Shown are results from the model based on binary receptor descriptors (A) and the model based on physicochemical description of amino acids of the transmembrane regions presumed to face in a direction opposite to the membrane (B) (see Methods for further details).

Model	R^2^	RMSE (log(M))	Q^2^	iR^2^	iQ^2^	eQ^2^
A	0.91	0.45	0.83	0.49	-0.21	0.84
B	0.91	0.46	0.82	0.46	-0.25	0.85

**Table 3:** Selectivity recognition map predicted from binary model. Selectivity recognition map for wild-type MC receptor pairs for α-MSH computed from the binary proteochemometric model. The contribution to the selectivity *S*^*u*^, log(M), for indicated segments S1–S5, was computed from the model using *Eq. 5*. Total selectivity represents the entire differences in affinity between receptor pairs computed from the model (see *Eq. 4*).

Segment	Contribution to selectivity *S*^*u*^, (log(M))					
	MC_1_/MC_3_	MC_1_/MC_4_	MC_1_/MC_5_	MC_3_/MC_4_	MC_3_/MC_5_	MC_4_/MC_5_
S1	1.14	1.07	1.04	-0.07	-0.09	-0.02
S2	0.39	0.91	1.15	0.52	0.77	0.24
S3	-0.15	0.33	0.44	0.49	0.60	0.11
S4	-0.07	0.54	0.62	0.61	0.69	0.08
S5	0.10	0.29	0.46	0.19	0.36	0.17
Total selectivity	1.40253	3.1389	3.71986	1.73638	2.31735	0.58095

**Table 4:** Predicted amino acid selectivity recognition map. Amino acid selectivity recognition map for MC receptors for α-MSH computed from the proteochemometric model based on the physicochemical description of amino acids of the transmembrane regions presumed to facing in the direction opposite to the membrane. The contributions to selectivity *S*^*u*^, log(M), of the indicated amino acid positions were computed from the model using *Eq. 5*. Total selectivity represents the entire difference in affinity between receptor pairs computed from the model (see *Eq. 4*). TM, transmembrane regions; SG, segments.

Amino Acids				TM	SG	Contribution to selectivity *S*^*u*^, (log(M))					
MC_1_	MC_3_	MC_4_	MC_5_			MC_1_/MC_3_	MC_1_/MC_4_	MC_1_/MC_5_	MC_3_/MC_4_	MC_3_/MC_5_	MC_4_/MC_5_
Glu37	Gln74	Gln43	Asp35	1	S1	0.23	0.23	0.18	0.00	-0.05	-0.05
Val38	Val75	Leu44	Met36	1	S1	0.00	0.11	0.12	0.11	0.12	0.00
Ser41	Lys78	Ser47	Ala39	1	S1	0.04	0.00	0.05	-0.04	0.01	0.05
Asp42	Pro79	Pro48	Val40	1	S1	0.21	0.21	0.21	0.00	0.00	0.00
Val60	Ile97	Ile66	Ile58	1	S1	0.48	0.48	0.48	0.00	0.00	0.00
Ile63	Val100	Ile69	Ile61	1	S1	0.07	0.00	0.00	-0.07	-0.07	0.00
Ile77	Leu114	Ile83	Val75	2	S2	-0.11	0.00	0.07	0.11	0.18	0.07
Ser83	Ala120	Ala89	Ala81	2	S2	0.14	0.14	0.14	0.00	0.00	0.00
Asn91	Asn128	Asn97	Ser89	2	S2	0.00	0.00	0.14	0.00	0.14	0.14
Val92	Ala129	Gly98	Ala90	2	S2	0.03	0.10	0.03	0.07	0.00	-0.07
Ala96	Ile133	Ile102	Ile94	2	S2	0.14	0.14	0.14	0.00	0.00	0.00
Leu99	Ala136	Thr105	Tyr97	2	S2	-0.01	0.05	0.06	0.06	0.07	0.01
Gln114	Gln151	Val119	Arg112	3	S2	0.00	0.08	0.11	0.08	0.11	0.03
Leu116	Met153	Ile121	Ile114	3	S2	-0.16	0.01	0.01	0.17	0.17	0.00
Ile120	Phe157	Ile125	Phe118	3	S2	-0.03	0.00	-0.03	0.03	0.00	-0.03
Thr124	Ile161	Ile129	Ile122	3	S2	0.14	0.14	0.14	0.00	0.00	0.00
Met128	Leu165	Leu133	Val126	3	S2	0.09	0.09	0.11	0.00	0.02	0.02
Leu129	Val166	Leu134	Val127	3	S2	-0.03	0.00	-0.03	0.03	0.00	-0.03
Leu132	Ile169	Ile137	Met130	3	S2	0.01	0.01	0.15	0.00	0.14	0.14
Gly136	Leu173	Leu141	Leu134	3	S2	0.14	0.14	0.14	0.00	0.00	0.00
Ala171	Cys208	Ala176	Phe169	4	S3	-0.09	0.00	0.04	0.09	0.13	0.04
Ser172	Cys209	Cys177	Cys170	4	S3	0.12	0.12	0.12	0.00	0.00	0.00
Phe175	Cys212	Ser180	Cys173	4	S3	0.02	0.11	0.02	0.09	0.00	-0.09
Ser176	Gly213	Gly181	Gly174	4	S3	0.12	0.12	0.12	0.00	0.00	0.00
Asp184	Glu221	Asp189	Glu182	5	S3	-0.07	0.00	-0.07	0.07	0.00	-0.07
Ala187	Met224	Ala192	Tyr185	5	S3	-0.08	0.00	-0.03	0.08	0.05	-0.03
Phe195	Met232	Met200	Met193	5	S3	0.12	0.12	0.12	0.00	0.00	0.00
Leu200	Met237	Leu205	Leu198	5	S3	-0.26	0.00	0.00	0.26	0.26	0.00
Met203	Met240	Met208	Leu201	5	S3	0.00	0.00	0.16	0.00	0.16	0.16
Leu243	Ile281	Leu247	Val240	6	S4	-0.07	0.00	0.63	0.07	0.70	0.63
Leu247	Leu285	Ile251	Leu244	6	S4	0.00	0.57	0.00	0.57	0.00	-0.57
Ile264	Ile302	Tyr268	Met261	6	S5	0.00	0.13	0.12	0.13	0.12	0.00
Val265	Ile303	Ile269	Leu262	6	S5	0.02	0.02	0.11	0.00	0.09	0.09
Ala285	Val323	Ile289	Ile282	7	S5	0.02	0.06	0.06	0.05	0.05	0.00
Ile288	Met326	Met292	Met285	7	S5	0.04	0.04	0.04	0.00	0.00	0.00
Ala291	Ser329	Ser295	Ser288	7	S5	0.04	0.04	0.04	0.00	0.00	0.00
Ile292	Val330	Ile296	Val289	7	S5	-0.01	0.00	-0.01	0.01	0.00	-0.01

Total selectivity				-		1.30	3.26	3.70	1.96	2.40	0.44

**Table 5:** Experimentally determined pK values of α-MSH for MC_4 _receptor mutants. Shown is the average pK ± SD determined in radioligand binding competition using [^125^I]NDP-MSH as radioligand. (The numbers of independent replicates are in brackets). The significance, p, is calculated using nonparametric Wilcoxon Rank Sum test [29] (see Methods for details), when compared with the wild-type MC_4 _receptor.

Name	pK_i _(log(nM)) ± SD (Repeats)	p
MC_4_	5.73 ± 0.32 (22)	-
Q43E/P48D	6.19 ± 0.14 (11)	1.3E-4
I66V	5.98 ± 0.26 (12)	9.8E-3
A89S	5.69 ± 0.14 (14)	6.4E-1
I102A	5.56 ± 0.30 (12)	1.8E-1
I129T	6.02 ± 0.15 (11)	4.1E-3
A135S	6.00 ± 0.15 (10)	1.0E-2
L141G	6.08 ± 0.15 (9)	3.0E-3
I251L	5.71 ± 0.36 (10)	7.0E-1
Y268I	5.88 ± 0.20 (14)	3.8E-2
Q43E/P48D/I66V	6.30 ± 0.17 (11)	1.5E-5
I66V/L141G	6.03 ± 0.20 (11)	4.6E-3
I129T/A135S	6.47 ± 0.51 (10)	2.3E-5
L141G/Y268I	6.01 ± 0.27 (12)	9.8E-3
Q43E/P48D/I66V/I129T/A135S	6.52 ± 0.17 (11)	1.4E-7
Q43E/P48D/I66V/I129T/A135S/L141G/Y286I	6.49 ± 0.31 (9)	1.8E-5
N240G/M241L/I245V	6.10 ± 0.10 (5)	1.1E-2
V253I/V255F/V256L	6.19 ± 0.16 (5)	4.3E-3
IL3	6.72 ± 0.14 (5)	1.7E-5
S4	6.98 ± 0.17 (5)	1.7E-5

**Table 6:** Description of peptides for proteochemometric modelling. The general sequence of the peptides used herein was: Ac-Ser-*Pos2*-Ser-*Pos4*-Gly-His-*Pos7*-Arg-Trp-Gly-Lys-Pro-Val-NH_2_. Amino acids in *Pos2*, *Pos4*, *Pos7*, and the corresponding description, were as shown.

Peptides	Sequence position			Descriptors		
	*Pos2*	*Pos4*	*Pos7*	***Y***	***F***	Y*F
α-MSH	Tyr	Met	Phe	-1	-1	1
NDP-MSH	Tyr	Nle	D-Phe	-1	1	-1
^125^I-NDP-MSH	^125^I-Tyr	Nle	D-Phe	1	1	1
